# Essential information on genetic testing methods that each clinician needs to know/understand

**DOI:** 10.1192/j.eurpsy.2021.157

**Published:** 2021-08-13

**Authors:** D. Coviello, V. Bizzarri, L. Nobili, M. Amore, K. Tammimies

**Affiliations:** 1 Laboratory Of Human Genetics, IRCCS Istituto Giannina Gaslini, Genoa, Italy; 2 Child Neuropsychiatric Unit, AUSL3-Liguria, Genoa, Italy; 3 Department Of Child And Adolescent Psychiatry, IRCCS Istituto Giannina Gaslini, Genoa, Italy; 4 Department Of Neuroscience, Rehabilitation, Ophthalmology, Genetics, Maternal And Child Health (dinogmi), Departimento di Neuroscienze, Università di Genova, Genoa, Italy; 5 Center Of Neurodevelopmental Disorders, Karolinska Institute, Stockholm, Sweden

**Keywords:** exome/genome analysis, Copy Number Variation (CNV), genetic testing, testing methods

## Abstract

Genetic testing is well established in many areas of clinical medicine, is increasingly used in clinical psychiatry and it becomes increasingly important to understand the scope and limitations of the different genetic tests applied. The recommended genetic work-up of patients with neurodevelopmental disorders (such as intellectual disability or autism spectrum disorders) includes conventional karyotyping (low resolution) able to detect chromosomal rearrangement and structural variants (>5Mb, 5 million-bp), testing for fragile X-Syndrome, screening for deletions and duplications down to 20 Kb by Comparative Genomic Hybridisation (CGH), able to detect Copy Number Variation (CNVs; gain or loss of genetic material compared to the reference genome). Sanger sequencing is used for mapping of single base pair genetic variants in single genes but unable to identify deletions or duplications. The more advanced Next Generation Sequencing (NGS) have enabled to detect variants in panels of 10-100 (or more) genes, or in all coding regions using Whole Exome Sequencing (WES; 23.000 genes). Whole Genome Sequencing (WGS) analysis enables also the detection of all size range and types of genetic variation including CNVs, trinucleotide repeats and translocations. All this led to an impressive change in interpreting genomic variants that need to be strictly linked to clinical information before it can be used by clinicians to improve diagnosis or care. Bioinformatic tools to annotate variants, predict their effects and select the genes and genomic regions of interest are needed to guide the clinical work followed with careful evaluation of the prioritized variants based on the clinical knowledge (https://www.cost.eu/actions/CA17130/#tabs|Name:overview).

**Disclosure:**

No significant relationships.

